# Inhibitory Effect and Mechanism of* Arctium lappa* Extract* on NLRP3* Inflammasome Activation

**DOI:** 10.1155/2018/6346734

**Published:** 2018-01-18

**Authors:** Young-Kyu Kim, Sushruta Koppula, Do-Wan Shim, Eun-Jung In, Su-Bin Kwak, Myong-Ki Kim, Sang-Hyeun Yu, Kwang-Ho Lee, Tae-Bong Kang

**Affiliations:** ^1^Department of Applied Life Science, Graduate School, Konkuk University, Chungju, Republic of Korea; ^2^Department of Biotechnology, College of Biomedical & Health Science, Research Institute of Inflammatory Diseases, 268 Chungwon-daero, Chungju, Republic of Korea; ^3^Department of Food Science and Engineering, Seowon University, Cheongju, Republic of Korea

## Abstract

*Arctium lappa (A. lappa)*, Compositae, is considered a potential source of nutrition and is used as a traditional medicine in East Asian countries for centuries. Although several studies have shown its biological activities as an anti-inflammatory agent, there have been no reports on* A. lappa* with regard to regulatory role in inflammasome activation. The purpose of this study was to investigate the inhibitory effects of* A. lappa* extract (ALE) on NLRP3 inflammasome activation and explore the underlying mechanisms. We found that ALE inhibited IL-1*β* secretion from NLRP3 inflammasome activated bone marrow derived macrophages but not that secreted by NLRC4 and AIM2 inflammasomes activation. Mechanistic studies revealed that ALE suppressed the ATPase activity of purified NLRP3 and reduced mitochondrial reactive oxygen species (mROS) generated during NLRP3 activation. Therefore, the inhibitory effect of ALE on NLRP3 inflammasome might be attributed to its ability to inhibit the NLRP3 ATPase function and attenuated the mROS during inflammasome activation. In addition, ALE significantly reduced the LPS-induced increase of plasma IL-1*β* in mouse peritonitis model. These results provide evidence of novel anti-inflammatory mechanisms of* A. lappa*, which might be used for therapeutic applications in the treatment of NLRP3 inflammasome-associated inflammatory disorders.

## 1. Introduction

Inflammasomes are cytosolic multimeric protein complexes composed of nucleotide-binding domain and leucine-rich-repeat-containing proteins (NLRs) such as NLRP3, NLRC4, or AIM2 (absent in melanoma 2), adapter protein ASC (apoptosis-associated speck-like protein containing a CARD), and caspase-1 [1]. Although each inflammasome is activated by different stimuli, inflammasomes serve as platforms for mediating the activation of caspase-1, which generates the biologically active cytokines IL-1*β* and IL-18. Active caspase-1 can also induce a kind of cell death known as pyroptosis through the cleavage of the cytosolic substrate gasdermin D [2]. NLRP3 is currently the well-characterized inflammasome and it is activated in response to various stimuli such as extracellular ATP, the potassium ionophore nigericin, and particulates such as silica crystals and asbestos [3–5]. Importantly, the NLRP3 inflammasome is also triggered by abnormal metabolites such as cholesterol crystals, monosodium urate crystals, and *β*-amyloid, which are implicated in the development of various metabolic disorders [6–8]. Therefore, the involvement of NLRP3 inflammasome in various diseases makes it a highly desirable therapeutic target.

The perennial herb family Burdock* (Arctium lappa, *Compositae) from the sunflower family is cultivated worldwide and has been used for centuries as a healthy and nutritive medicinal food in East Asian countries. In addition, its various botanical parts have been traditionally used for burns, tumors, eczema, gout, and hepatitis, indicating the existence of anti-inflammatory activity. Many recent studies have revealed that* A. lappa* extracts and its bioactive constituents inhibit several inflammation processes, such as inducible nitric oxide synthase (iNOS), proinflammatory cytokines TNF, and IL-6 expression [9]. Although numerous studies have shown the anti-inflammatory effect of* A. lappa*, none have reported its efficacy on inflammasome activation. Therefore, the present study was designed to investigate the inhibitory potential and underlying mechanism of the extracts of* A. lappa* leaves and stems (ALE) on inflammasome activation by examining its effects on IL-1*β* secretion and modification of the inflammasome-related molecules upon stimulation with various inflammasome stressors.

## 2. Materials and Methods

### 2.1. Plant Material

The powder of a methanol extract (ALE) from the leaves and stems of* A. lappa* (catalog number: 011-076) was obtained from the plant extract bank at the Korea Research Institute of Bioscience and Biotechnology (KRIBB) (Daejeon, Korea). The powder was then dissolved in dimethyl sulfoxide (DMSO) and diluted with cell culture media immediately before use. The final concentration of DMSO in the cell culture media was maintained below 0.1%.

### 2.2. Reagents and Antibodies

IL-1*β* and TNF-*α* ELISA kits were purchased from eBioscience (San Diego, CA, USA) or R&D Systems (Minneapolis, MN, USA). Silica crystals (nano-SiO_2_), nigericin, poly(dA:dT), flagellin, and carbobenzoxy-valyl-alanyl-aspartyl-[O-methyl]-fluoromethylketone (zVAD-FMK) were purchased from InvivoGen (San Diego, CA, USA). LPS (0111:B4), ATP, L-cysteine, glutathione, and forskolin were obtained from Sigma-Aldrich (St. Louis, MO, USA). H89 was purchased from Alexis Biochemicals (San Diego, CA, USA). Lipofectamine 2000 and disuccinimidyl suberate (DSS) were acquired from Thermo Fisher Scientific (Rockford, IL, USA). G5 was purchased from EMD Millipore (Bedford, MA, USA). The lactic dehydrogenase (LDH) detection kit was from Dogen EINNOTEC (Dae Jeon, Korea). MCC950 was purchased from AdipoGen (San Diego, CA, USA). The following antibodies were used for western blotting: anti IL-1*β*, (AF-401-NA, R&D Systems, Minneapolis, MN, USA); NLRP3 (Cryo-2), ASC (AL-177), and caspase-1 (AG-20B-0042-C100) from AdipoGen (San Diego, CA, USA); ASC (N-15), caspase-1 (SC-514), and *β*-actin from Santa Cruz Biotechnology (Dallas, TX, USA).

### 2.3. Cell Culture and Stimulation

Bone marrow cells from C57BL/6 mice (7 weeks old) were harvested by flushing the femurs and tibias with sterile PBS. These cells were cultured in RPMI 1640 supplemented with 10% fetal bovine serum (FBS), 30% L929 cell conditioned medium (LCM), 100 *μ*M 2-mercaptoethanol, 100 unit/ml of penicillin, and 100 *μ*g/ml of streptomycin. On day three of culture, the same volume of culture medium was added and on day six, 50% of the cultured medium was replaced with fresh medium. On day seven, the nonadherent cells were removed and the adherent cells were detached and seeded at a density of 1 × 10^6^ cells into 6-well plates. Unless otherwise specified, the activation of inflammasomes was first primed with LPS (100 ng/mL) for 3 h, then the medium was replaced with Opti-MEM, and the cells were preincubated with ALE or inhibitors, zVAD or KCl (150 mM) for 30 min. The cells were then stimulated with ATP (5 mM) and nigericin (10 *μ*M) for 1 h and silica crystals (150 *μ*g/ml) for 3 h; and flagellin (1.5 *μ*g/ml) or poly(dA:dT) (2 *μ*g/ml) was transfected for 3 h or 1 h, respectively.

### 2.4. ASC Oligomerization Assay

Isolation of the Triton X-100 insoluble fraction and chemical crosslinking using DSS were conducted as previously described with some modifications [10, 11]. Cells were lysed with lysis buffer A [50 mM Tris-HCl (pH 7.5), 0.5% Triton X-100, and protease inhibitor cocktail] for 20 min on ice. Soluble lysates and precipitated pellets prepared by centrifugation (600 ×g, 15 min at 4°C) were used as the Triton X-100 soluble fractions and the Triton-insoluble fractions, respectively. For the ASC oligomerization assay, the Triton X-100 insoluble precipitates were washed twice with cold PBS and crosslinked in 2 mM DSS/PBS for 30 min at room temperature. The pellets were harvested by centrifugation for 15 min at 13,000 rpm and dissolved in SDS sample buffer for further western blot analysis.

### 2.5. Assessment of ASC Speck Formation

The cells (3 × 10^5^ cells/well) were seeded on coverslips in 12-well plates and were stimulated with the indicated stimuli. The cells were then washed with cold PBS and fixed in 4% (v/v) paraformaldehyde for 20 min at 4°C and followed by incubation in acetone for 10 min at −20°C after rinsing with PBS. Cells were washed twice with PBS and blocked with 10% (v/v) horse serum in PBS with Tween 20 (PBST) for 1 h at room temperature before incubation with rabbit anti-ASC (1 : 200) antibody for 2 h at room temperature. Cells were washed with PBST and incubated with Cy3-conjugated anti-rabbit IgG (1 : 300) for 1 h at room temperature in the dark, and then the cell nuclei were stained with DAPI (1 : 50000).

### 2.6. *In Vivo* Challenge with LPS

C57BL/6 mice were purchased from OriGent Bio (Sung Nam, Korea). They were housed in groups of five under standard conditions (temperature 22 ± 2°C, humidity 55 ± 5%, 12 h light/dark cycle) with food and distilled water ad libitum. All experiments were performed under the guidelines of the Konkuk University Animal Care Committee, Korea (license number: KU17050). The animals were allowed to adapt to the laboratory environment for five to seven days before the experiments were conducted. For the LPS-induced systemic inflammation experiments, 7~9-week-old age- and sex-matched mice were given 20 or 40 mg/kg ALE or vehicle control intraperitoneally (*i.p.*), 2 h and 12 h prior to LPS injection (20 mg/kg). Blood samples were collected 2 h after the LPS challenge.

### 2.7. Enzyme-Linked Immunosorbent Assay (ELISA)

Measurement of cytokines (IL-1*β* and TNF-*α*) was determined using an ELISA kit (eBioscience or R&D Systems) according to the manufacturers' instructions. The color generated was measured at 450 nm using a spectrophotometric microplate reader (Molecular Devices Corp., Sunnyvale, CA, USA).

### 2.8. Immunoblot Analysis

Immunoblotting was performed using a standard system as previously described [12, 13]. Protein lysates were prepared in RIPA buffer [50 mM Tris-HCl (pH 7.5), 1% Nonidet P-40, 150 mM sodium chloride, 0.5% sodium deoxycholate, 0.1% sodium dodecyl sulfate (SDS), and protease inhibitor cocktails] or Triton X-100 lysis buffer [50 mM Tris-HCl (pH 7.5), 1% Triton X-100, 2 mM EDTA, 150 mM sodium chloride, and protease inhibitor cocktails] and were processed by SDS-PAGE, blotted to a nitrocellulose membrane, and sequentially probed with primary antibodies and horseradish peroxidase- (HRP-) conjugated antibody. An enhanced chemiluminescence detection kit (Thermo Fisher Scientific) was used to visualize the secondary antibodies using the Luminescent Image Analyzer (LAS-3000; Fujifilm, Tokyo, Japan).

### 2.9. *In Vitro* Cell Viability Assays

Bone marrow derived macrophages (BMDMs) were treated with the indicated concentrations of ALE for 6 h and then incubated with MTT solution (0.5 mg/ml) 2 h at 37°C. Then, insoluble formazan was solubilized with DMSO (100 *μ*l/well) and the optical density was measured at 550 nm. The cell viability was calculated as (viable cells) % = (OD of drug-treated sample/OD of untreated sample) × 100.

### 2.10. Measurement of ATPase Activity of NLRP3

ATPase activity of NLRP3 was measured as described [14]. Human recombinant NLRP3 (BPS Bioscience, San Diego, CA, USA) was preincubated with the DMSO or ALE for 20 min at 37°C in reaction buffer. The reaction buffer contained 20 mM Tris-HCl pH 7.8, 133 mM NaCl, 20 mM MgCl_2_, 3 mM KCl, and 0.56 mM EDTA. Then, ultra-pure-ATP (250 *μ*M) was added, and the mixtures were further incubated for 40 min at 37°C. The hydrolysis of ATP by NLRP3 was determined by measurement of ADP using ADP-Glo Kinase Assay (Promega, Madison, WI, USA) following the manufacturers' instruction.

### 2.11. Measurement of Mitochondrial Reactive Oxygen Species (mROS)

The mROS levels were determined by staining cells with MitoSOX (Invitrogen, Carlsbad, CA, USA). Cells were incubated with 2.5 *μ*M MitoSOX for 30 min at 37°C, washed with prewarmed PBS solution, and resuspended in cold PBS containing 2% FBS and Topro-3 (Thermo Fisher Scientific. Rockford, IL, USA) for FACS analysis.

### 2.12. Fingerprint Analysis of ALE Using High-Performance Liquid Chromatography (HPLC)

ALE was dissolved in 80% methanol and centrifuged for 5 min at 3,000 rpm. The supernatant was used as the analytical sample for the determination. The components of ALE were determined with the Prominence LC-20A (Shimadzu, Kyoto, Japan). The HPLC system was equipped with a CMA-20A communications bus module, LC 20AD liquid chromatograph, SPD-M20A diode array detector, SIL-20AC autosampler, DGU-20A3 degasser, and CTO-20AC column oven. HPLC analysis was conducted using a Shim-pack VP-ODS (3 × 75 mm, 2.2 *μ*m, Shimadzu, Japan) The flow rate of the mobile phase was 0.15 ml/min. The chromatogram was monitored at 330 nm. The injection volume was 10 *μ*L. The column temperature was maintained at 30°C. The mobile phase, consisting of 100% acetonitrile (*A*) and water containing 0.1% formic acid (*B*), was run with the gradient programs shown in Table 1. The LC/MS-IT-TOF was run with the gradient programs shown in Table 1. The LC/MS-IT-TOF was operated with a nebulizer gas flow rate of 1.5 ml/min, detector voltage of 1.53 kV, and probe voltage of 450 kV. The mass range (*m*/*z*) was 100–1500 amu.

### 2.13. Statistical Analysis

Data were presented as mean ± the standard deviation (SD) or the standard error of the mean (SEM). Statistical analyses were assessed by one-way ANOVA, followed by the Dunnett's post hoc test using GraphPad PRISM5 software (San Diego, CA, USA) and the values of *p* < 0.05 were considered statistically significant.

## 3. Results

### 3.1. ALE Inhibits NLRP3 Inflammasome Mediated IL-1*β* Secretion

**Figure 1:**
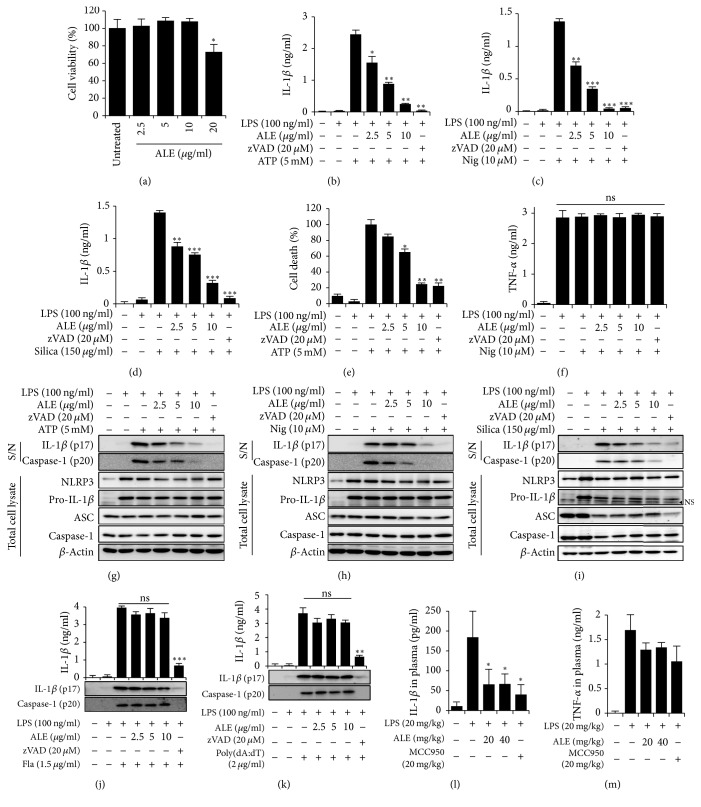
Inhibitory effects of ALE on NLRP3 inflammasome mediated IL-1*β* secretion* in vitro *and* in vivo*. (a) BMDMs were treated with the indicated concentration of ALE for 6 h. Cell viability was measured by MTT assay. LPS-primed BMDMs were pretreated with ALE or zVAD at an indicated concentration for 1 h and then stimulated with ATP (b, e, and g); nigericin (Nig.) (c, f, and h) for 1 h; and silica crystals (silica) (d and i) for 3 h. IL-1*β* (b–d) and TNF-*α* (f) concentrations in the culture supernatant were measured by ELISA. (e) Cytotoxicity of ALE was measured with LDH release in the culture supernatants. (g~i) Culture supernatants (S/N) and cell lysates from BMDMs pretreated with or without sample and primed with LPS and indicated stimulators were analyzed by immunoblotting. LPS-primed BMDMs were treated with ALE or zVAD at an indicated concentration for 1 h, and flagellin (Fla) (j) or poly(dA:dT) (k) was delivered into cytosol through lipofectamine 2000. Culture supernatants were analyzed by immunoblotting. (l and m) C57BL/6 mice were given intraperitoneal injections of ALE or a NLRP3 specific inhibitor, MCC950, 2 h and 12 h prior to LPS injection (20 mg/kg). Blood samples were collected 2 h after the LPS challenge and the concentrations of IL-1*β* (l) and TNF-*α* (m) in plasma were measured by ELISA. The data represent the mean ± SEM of three independent experiments performed in triplicate. ^*∗*^*p* < 0.05, ^*∗∗*^*p* < 0.01, and ^*∗∗∗*^*p* < 0.001 compared with LPS plus stimuli.

To determine whether ALE has an inhibitory effect on inflammasome activation, LPS-primed BMDMs were pretreated with ALE at nontoxic concentrations (Figure 1(a)) before stimulation with NLRP3 agonists, ATP, nigericin, and silica crystals. A pan-caspase inhibitor (zVAD-Fmk) was used as a positive control to inhibit caspase-1 activation. As indicated in Figures 1(b)–1(d), the ELISA results showed that ALE at 2.5~10 *μ*g/ml was able to suppress the release of IL-1*β* induced by NLRP3 inflammasome activators. It has been reported that caspase-1-mediated programmed cell death, called pyroptosis, occurs during inflammasome activation [10]. Therefore, we also examined the effect of ALE on inflammasome activation-induced cell death by measuring the release of lactate dehydrogenase (LDH) in the culture supernatants. As expected, the administration of ALE inhibited ATP-induced pyroptosis in a dose dependent manner (Figure 1(e)). Importantly, under the same conditions, the release of TNF-*α* was not suppressed by ALE treatment, suggesting that ALE might inhibit the activation step in inflammasomes rather than the LPS-induced priming step (Figure 1(f)).

Inflammasomes are multiprotein complexes that activate caspase-1, leading to processing and secretion of the IL-1 family [15]. Therefore, to determine whether the inhibition of IL-1*β* release by ALE was caused by the control of inflammasome activation, the impact of ALE treatment on the cleavage of pro-caspase-1 and pro-IL-1*β* was examined. Consistent with the ELISA results, ALE suppressed the processing of IL-1*β* as well as the activation of caspase-1 (Figures 1(g)–1(i)). Notably, the treatment of ALE did not affect the expression of NLRP3 inflammasome components, NLRP3, caspase-1, pro-IL-1*β*, or ASC in cell lysates.

To examine whether the inhibition of inflammasomes by ALE is specific to the NLRP3 inflammasome, its effects on NLRC4 and AIM2 inflammasome activation were assessed. Cytosolic bacterial flagellin and dsDNA can trigger NLRC4 and AIM2 inflammasome activation, respectively [16, 17]. Therefore, we assessed the effect of ALE on the processing and release of IL-1*β* and caspase-1 cleavage in BMDMs upon stimulation with flagellin or poly(dA:dT). In contrast to its inhibitory effect on NLRP3 agonist-mediated caspase-1 and IL-1*β* processing, ALE had no effect on the flagellin- or poly(dA:dT)-induced caspase-1 cleavage (Figures 1(j)–1(k)), which indicated that ALE inhibits processing and release of both IL-1*β* and caspase-1 through the control of NLRP3 inflammasome activation, but not NLRC4 or AIM2 inflammasome activation.

To check the* in vivo* relevance of inhibitory action of ALE on IL-1*β* release, we examined whether ALE treatment could suppress the elevated plasma level of IL-1*β* in LPS-induced mouse peritonitis model. In parallel, NLRP3-specific inhibitor, MCC950 [18], was treated as a positive control. In accordance with the* in vitro* results, ALE administration significantly reduced IL-1*β* level in plasma (Figure 1(l)). However, the elevation of TNF-*α* in plasma in the same condition was not affected by ALE treatment (Figure 1(m)).

### 3.2. ALE Inhibits NLRP3-Induced ASC Translocation, Oligomerization, and Speck Formation

**Figure 2:**
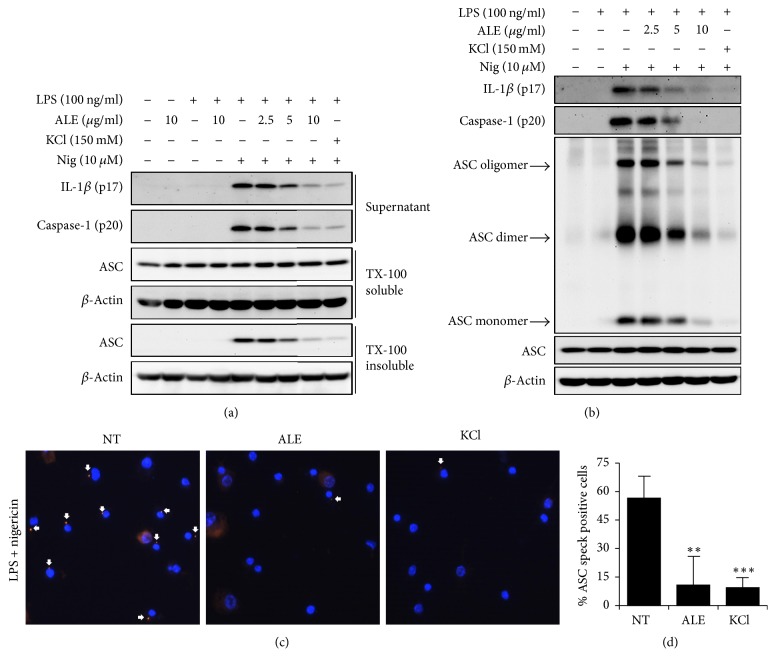
Inhibitory effects of ALE on ASC translocation and oligomerization. LPS-primed BMDMs were pretreated with ALE or KCl for 30 min, before stimulation with nigericin (Nig). (a) Cleaved IL-1*β* (p17) and caspase-1 (p20) in culture supernatant and ASC in TX-soluble and insoluble fractions were analyzed by immunoblotting. (b) Immunoblotting showing ASC oligomerization in crosslinked lysates of BMDMs. (c) Representative ASC speck (arrowed) images upon stimulation with LPS plus nigericin. Nuclei are depicted in blue (DAPI) and ASC in red. (d) Quantification of cells with ASC speck presented the % of positive cells. At least five fields and more than 200 cells were counted for each condition. KCl was used as a positive control. The data represent the mean ± SEM of three independent experiments. ^*∗∗*^*p* < 0.01 and ^*∗∗∗*^*p* < 0.001 compared with LPS plus nigericin.

To investigate the action mechanism of ALE in the inhibition of inflammasome activation, we analyzed the upstream molecular events of caspase-1 and IL-1*β* processing. Upon inflammasome activation, the ASC molecule has been shown to be redistributed to the detergent insoluble fraction and form the oligomerization and subsequent assembly of ASC speck [10, 11]. Therefore, we examined whether ALE affects ASC translocation, oligomerization, and speck formation upon stimulation with NLRP3 agonists and a potassium channel inhibitor, KCl, was used as positive to inhibit inflammasome activation. As shown in Figure 2(a), nigericin treatment induced the redistribution of ASC and its oligomerization in BMDMs. The pretreatment of ALE significantly and dose-dependently prevented the translocation of ASC to the detergent insoluble part and its oligomerization in a dose dependent manner (Figures 2(a) and 2(b)). Furthermore, ALE substantially reduced the formation of ASC speck in BMDMs induced by nigericin (Figures 2(c) and 2(d)). Collectively, these data suggested that ALE inhibits NLRP3 inflammasome by the prevention of ASC redistribution, oligomerization, and speck formation, which are required for NLRP3 inflammasome activation.

### 3.3. ALE Inhibits NLRP3 Inflammasome Activation through the Inhibition of ATPase Activity and the Suppression of Mitochondrial ROS Generation

**Figure 3:**
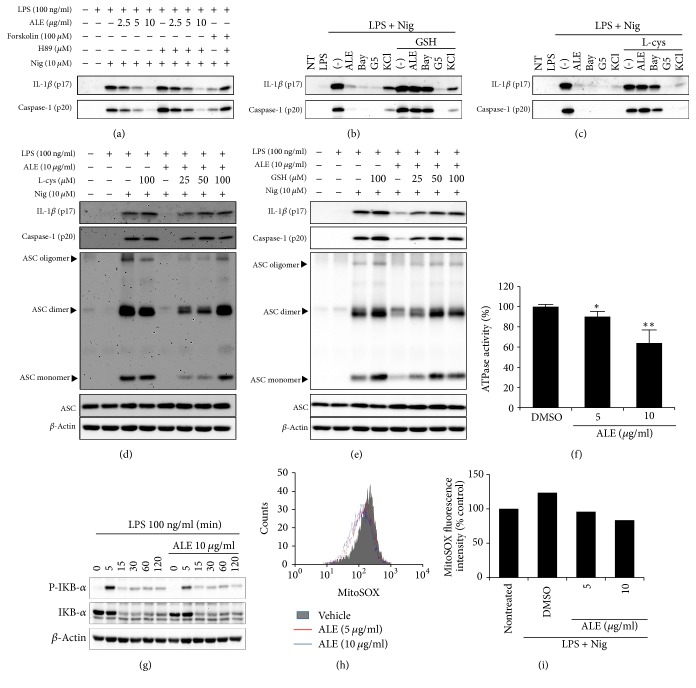
Alkylation-mediated NLRP3 inflammasome inhibition of ALE. (a) Mature IL-1*β* and active caspase-1 in culture supernatants of LPS-primed BMDMs treated with ALE or forskolin in the presence or absence of H89 for 30 min, followed by nigericin (Nig) for 1 h, were immunoblotted. (b–g) LPS-primed BMDMs were treated with ALE, Bay11-7082 (Bay), G5, or KCl in the presence or absence of L-cysteine (L-cys) (b and d) or glutathione (GSH) (c and e) for 15 min, followed by nigericin for 1 h, and culture supernatants and crosslinked lysates were analyzed by immunoblotting. (f) ATPase activity of NLRP3 in the presence or absence of ALE was determined by luminescence by using the ADP-Glo assay. The data represent the mean ± SD of two independent experiments. ^*∗*^*p* < 0.05 and ^*∗∗*^*p* < 0.01 compared with vehicle treated control (g) BMDMs were treated with ALE for 30 min before stimulation with LPS for the indicated time, and phospho-I*κ*B-*α* and I*κ*B-*α* in cell lysates were analyzed by immunoblotting. (h and i) The LPS-primed BMDMs were stimulated nigericin in the presence or absence of ALE. Levels of mitochondrial ROS in BMDM were analyzed by MitoSOX labeling. (h) The representative flow cytometric histogram and (i) quantitative analysis of mean fluorescence intensities of two independent experiments are shown.

ALE showed inhibitory activity on the NLRP3-mediated inflammasome, but not on NLRC4- and AIM2-mediated inflammasomes. Since ASC is known to be involved in the activation of both NLRP3 and AIM2 inflammasomes, mere inhibition of ASC oligomerization alone cannot explain how ALE specifically inhibits the NLRP3 inflammasome. We hypothesized that the inhibition of NLRP3 inflammasome by ALE might be through the targeting of NLRP3 itself or NLRP3 activation-associated upstream or downstream molecules. It has been reported that NLRP3 inflammasome activation can be modulated by the posttranslational regulation of NLRP3, such as phosphorylation, ubiquitination, and alkylation [19–22]. A protein kinase A- (PKA-) induced phosphorylation and ubiquitination of NLRP3 has been identified as a negative regulatory mechanism in NLRP3 inflammasome activation and a PKA inhibitor, H89, is known to counteract the suppressive effect of PKA on NLRP3 induced inflammasome activation [19, 20]. Recent studies showed that cysteine(s) modification of NLRP3 possesses a suppressive effect on inflammasome activation through the inhibition of NLRP3 recruitment and ASC oligomerization. Furthermore, excess amount of free L-cysteine or glutathione (GSH) prevented the suppressive effect by cysteine modification [14, 22, 23]. We, therefore, treated cells with various inhibitors combined with ALE to investigate if ALE has an impact on the modification of NLRP3. Consistent with the previous report [20], H89 impaired the inhibitory effect of forskolin-induced inflammasome activation but did not affect the inhibitory effect of ALE (Figure 3(a)), indicating that the inhibitory activity of ALE on NLRP3 activation is not through the PKA signaling pathway. However, the preincubation of L-cysteine or GSH with ALE dose-dependently abrogated the inhibitory function of ALE on IL-1*β* release, caspase-1 activation, and ASC oligomerization (Figures 3(d) and 3(e)). These results indicated that ALE might induce cysteine modification on protein(s) associated with NLRP3 inflammasome signaling pathway, which negatively regulates NLRP3 inflammasome activation. To verify the specific effect of L-cysteine and GSH on cysteine modification, Bay11-7082 [22], which inhibits inflammasome activation through the cysteine modification or G5 (deubiquitination inhibitor) [21], and KCl (potassium efflux inhibitor) [24] were used as experimental controls. As expected, although all inhibitors suppressed the release of IL-1*β* and caspase-1 from BMDMs upon stimulation, only Bay11-7082 diminished the inhibitory activity of ALE on the maturation of IL-1*β* and caspase-1 (Figures 3(b) and 3(c)).

Recent reports showed that the ATPase activity of NLRP3 is indispensable for the ASC oligomerization [23, 25]. Thereby, we tested whether ALE can inhibit ATPase activity of NLRP3. As shown in Figure 3(f), ALE suppressed the ATPase activity of NLRP3 in a dose dependent manner.

It has been shown that some anti-inflammasome drugs are also able to modify cysteine residue of IKK*β* and P65, which result in the suppression of NF-*κ*B activation [26–29]. Therefore, we explored whether ALE might target IKK*β* to inhibit NF-*κ*B activation. As shown in Figure 3(g), ALE could not affect LPS-induced I*κ*B-*α* phosphorylation and degradation at the same concentration that provides inhibitory activity on NLRP3 inflammasome activation. Therefore, these data suggest that the inhibitory effect of ALE was not attributed to the suppression of NF-*κ*B activation.

It has been known that mitochondrial ROS is a NLRP3-specific trigger for inflammasome activation [30]. Since the extracts of* Arctium lappa* has been shown to possess antioxidant activity [9], we also examined whether ALE could lower mROS level during NLRP3 inflammasome activation. As shown in Figures 3(h) and 3(i), ALE suppressed the generation of mROS induced by nigericin with LPS, suggesting that the antioxidant activity of ALE also partially contributes to its inhibitory effect on NLRP3 inflammasome activation.

### 3.4. HPLC Analysis of ALE

**Figure 4:**
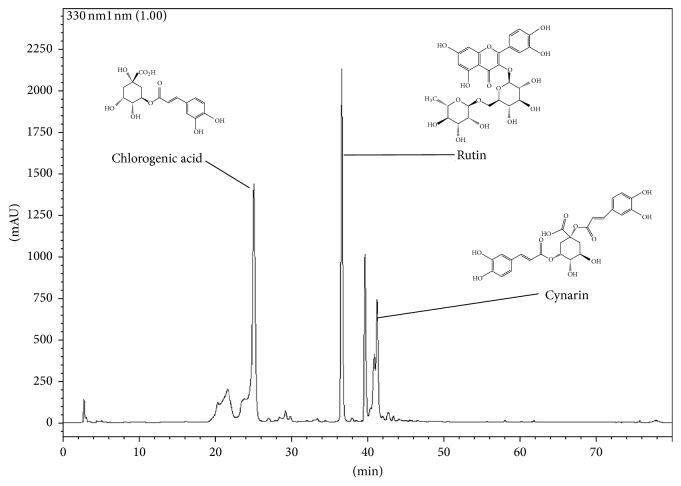
Fingerprinting of ALE. The components of ALE were determined using an HPLC system. ALE was dissolved in 80% methanol and applied to the HPLC system (Shim-pack VP-ODS column) at a flow rate of 0.15 ml/min.

The typical HPLC pattern of ALE is shown in Figure 4. The analysis revealed that ALE included chlorogenic acid, rutin, and cynarin as major peaks.

## 4. Discussion

Inflammasomes regulate the activity of caspase-1, and its activation causes the maturation and release of proinflammatory cytokines such as IL-1*β* or IL-18. Although this process is necessary for host defense against insults, its inappropriate activation contributes to the development of various autoimmune and inflammatory diseases [1]. Among several of the inflammasomes, NLRP3 inflammasome is relatively well studied and its activation is linked to age-related metabolic diseases and autoinflammatory diseases [31, 32]. Therefore, many efforts have been made to find inhibitors that can control NLRP3 inflammasome activation from various sources such as small molecules, interferon, autophagy inducers, and microRNA [18, 33–35]. In recent years, much attentions have been given to drug discovery from natural sources and their active constituents that can act as specific NLRP3 inflammasome inhibitor and many have been reported from others and our laboratory [36–38]. In our continuous search of specific inhibitors of NLRP3 inflammasome, we identified that the extracts of* A. lappa* leaves and stems suppressed the release of IL-1*β* through NLRP3 inflammasome activation induced by typical agonists, such as ATP, nigericin, and silica crystals. In addition, the inhibition of IL-1*β* release was further confirmed in the plasma of LPS-injected mice. Moreover, subcellular location, trafficking, oligomerization, and speck formation of adaptor molecule ASC were inhibited by ALE treatment during NLRP3 inflammasome activation. Interestingly, ALE did not inhibit flagellin- or poly(dA:dT)-induced inflammasome activation, indicating that ALE is specific for NLRP3 inflammasome and suggesting that ALE might target NLRP3 itself or NLRP3 specific signaling pathway.

Recent reports indicated that NLRP3 modifications such as ATPase activity and mitochondrial ROS (mROS) can regulate NLRP3-mediated inflammasome activation without affecting other types of inflammasomes [19–22, 25, 30]. Therefore, we assessed the impact of ALE on the modification of NLRP3, its ATPase function, and mROS induction during inflammasome activation. Indeed, the results showed that the anti-inflammasome activity of ALE was abrogated by the pretreatment of L-cysteine or free glutathione, indicating that the inhibitory effect of ALE on NLRP3 inflammasome might result from its ability to modify cysteine residue on a protein associated NLRP3 inflammasome signaling.

However, from our current data, it is not clear whether ALE suppress the NLRP3 inflammasome activation through the modification of NLRP3 itself or other proteins associated with NLRP3 function. Furthermore, ALE inhibits the ATPase activity of purified NLRP3 directly and attenuated the mROS level in cells, which suggests that, in addition to its ability to regulate the function of NLRP3, the antioxidative activity of ALE might also contribute in suppressing the NLRP3 inflammasome activation.

Earlier studies have shown that* A. lappa* as well as its components, arctigenin and arctiin, exhibited anti-inflammatory activities. Further, the active constituents identified in the ALE extract by HPLC fingerprinting showed distinct peaks of chlorogenic acid, rutin, and cynarin. Some of these compounds have been well established as possessing anti-inflammatory properties [39–41]. These compounds might act individually or in combination to deliver synergistic effect in inhibition of NLRP3 inflammasome activation. However, further studies on the isolation and identification of the active components present in ALE and its in-depth action mechanism on specific targets at molecular levels are required to better understand the mechanism of action of ALE.

In conclusion, our results provide a novel inflammasome-associated mechanism for* A. lappa* potential anti-inflammatory effects and might be developed as a therapeutic application in the treatment of NLRP3 inflammasome-associated inflammatory disorders.

## Figures and Tables

**Table 1:** Mobile phase condition of the HPLC.

Time (min)	*A*% (acetonitrile)	*B*% (0.1% formic acid)
0	5	95
5	5	95
20	15	85
30	20	80
50	40	60
55	90	10
60	90	10
61	5	95
70	5	95

## References

[1] Schroder K., Tschopp J. (2010). The inflammasomes. *Cell*.

[2] Shi J., Zhao Y., Wang K. (2015). Cleavage of GSDMD by inflammatory caspases determines pyroptotic cell death. *Nature*.

[3] Pelegrin P., Surprenant A. (2006). Pannexin-1 mediates large pore formation and interleukin-1*β* release by the ATP-gated P2X7 receptor. *EMBO Journal*.

[4] Pelegrin P., Surprenant A. (2007). Pannexin-1 couples to maitotoxin- and nigericin-induced interleukin-1*β* release through a dye uptake-independent pathway. *The Journal of Biological Chemistry*.

[5] Dostert C., Pétrilli V., van Bruggen R., Steele C., Mossman B. T., Tschopp J. (2008). Innate immune activation through Nalp3 inflammasome sensing of asbestos and silica. *Science*.

[6] Duewell P., Kono H., Rayner K. J. (2010). NLRP3 inflammasomes are required for atherogenesis and activated by cholesterol crystals. *Nature*.

[7] Martinon F., Pétrilli V., Mayor A., Tardivel A., Tschopp J. (2006). Gout-associated uric acid crystals activate the NALP3 inflammasome. *Nature*.

[8] Schnaars M., Beckert H., Halle A. (2013). Assessing *β*-amyloid-induced NLRP3 inflammasome activation in primary microglia. *Methods in Molecular Biology*.

[9] Chan Y.-S., Cheng L.-N., Wu J.-H. (2011). A review of the pharmacological effects of Arctium lappa (burdock). *Inflammopharmacology*.

[10] Fernandes-Alnemri T., Wu J., Yu J.-W. (2007). The pyroptosome: a supramolecular assembly of ASC dimers mediating inflammatory cell death via caspase-1 activation. *Cell Death & Differentiation*.

[11] Hara H., Tsuchiya K., Kawamura I. (2013). Phosphorylation of the adaptor ASC acts as a molecular switch that controls the formation of speck-like aggregates and inflammasome activity. *Nature Immunology*.

[12] Duojiao N., Peng X., Gallagher S. (2016). Immunoblotting and immunodetection. *Current Protocols in Molecular Biology*.

[13] Towbin H., Staehelin T., Gordon J. (1979). Electrophoretic transfer of proteins from polyacrylamide gels to nitrocellulose sheets: procedure and some applications. *Proceedings of the National Acadamy of Sciences of the United States of America*.

[14] Cocco M., Miglio G., Giorgis M. (2016). Design, synthesis, and evaluation of acrylamide derivatives as direct NLRP3 inflammasome inhibitors. *ChemMedChem*.

[15] Martinon F., Burns K., Tschopp J. (2002). The inflammasome: a molecular platform triggering activation of inflammatory caspases and processing of proIL-*β*. *Molecular Cell*.

[16] Franchi L., Amer A., Body-Malapel M. (2006). Cytosolic flagellin requires Ipaf for activation of caspase-1 and interleukin 1*β* in salmonella-infected macrophages. *Nature Immunology*.

[17] Hornung V., Ablasser A., Charrel-Dennis M. (2009). AIM2 recognizes cytosolic dsDNA and forms a caspase-1-activating inflammasome with ASC. *Nature*.

[18] Coll R. C., Robertson A. A., Chae J. J. (2015). A small-molecule inhibitor of the NLRP3 inflammasome for the treatment of inflammatory diseases. *Nature Medicine*.

[19] Mortimer L., Moreau F., MacDonald J. A., Chadee K. (2016). NLRP3 inflammasome inhibition is disrupted in a group of auto-inflammatory disease CAPS mutations. *Nature Immunology*.

[20] Guo C., Xie S., Chi Z. (2016). Bile acids control inflammation and metabolic disorder through inhibition of NLRP3 inflammasome. *Immunity*.

[21] Py B. F., Kim M.-S., Vakifahmetoglu-Norberg H., Yuan J. (2013). Deubiquitination of NLRP3 by BRCC3 critically regulates inflammasome activity. *Molecular Cell*.

[22] Juliana C., Fernandes-Alnemri T., Wu J. (2010). Anti-inflammatory compounds parthenolide and Bay 11-7082 are direct inhibitors of the inflammasome. *The Journal of Biological Chemistry*.

[23] Duncan J. A., Bergstralh D. T., Wang Y. (2007). Cryopyrin/NALP3 binds ATP/dATP, is an ATPase, and requires ATP binding to mediate inflammatory signaling. *Proceedings of the National Acadamy of Sciences of the United States of America*.

[24] Muñoz-Planillo R., Kuffa P., Martínez-Colón G., Smith B. L., Rajendiran T. M., Núñez G. (2013). K^+^ efflux is the common trigger of NLRP3 inflammasome activation by bacterial toxins and particulate matter. *Immunity*.

[25] Shim D., Shin W., Yu S. (2017). BOT-4-one attenuates NLRP3 inflammasome activation: NLRP3 alkylation leading to the regulation of its ATPase activity and ubiquitination. *Scientific Reports*.

[26] García-Piñeres A. J., Castro V., Mora G. (2001). Cysteine 38 in p65/NF-*κ*B plays a crucial role in DNA binding inhibition by sesquiterpene lactones. *The Journal of Biological Chemistry*.

[27] García-Piñeres A. J., Lindenmeyer M. T., Merfort I. (2004). Role of cysteine residues of p65/NF-*κ*B on the inhibition by the sesquiterpene lactone parthenolide and N-ethyl maleimide, and on its transactivating potential. *Life Sciences*.

[28] Hehner S. P., Hofmann T. G., Droge W., Schmitz M. L. (1999). The antiinflammatory sesquiterpene lactone parthenolide inhibits NF-kappa B by targeting the I kappa B kinase complex. *Journal of immunology*.

[29] Kwok B. H. B., Koh B., Ndubuisi M. I., Elofsson M., Crews C. M. (2001). The anti-inflammatory natural product parthenolide from the medicinal herb Feverfew directly binds to and inhibits I*κ*B kinase. *Chemistry & Biology*.

[30] Zhou R., Yazdi A. S., Menu P., Tschopp J. (2011). A role for mitochondria in NLRP3 inflammasome activation. *Nature*.

[31] Broz P., Dixit V. M. (2016). Inflammasomes: Mechanism of assembly, regulation and signalling. *Nature Reviews Immunology*.

[32] Guo H., Callaway J. B., Ting J. P.-Y. (2015). Inflammasomes: mechanism of action, role in disease, and therapeutics. *Nature Medicine*.

[33] Guarda G., Braun M., Staehli F. (2011). Type I interferon inhibits interleukin-1 production and inflammasome activation. *Immunity*.

[34] Shi C. S., Shenderov K., Huang N. N. (2012). Activation of autophagy by inflammatory signals limits IL-1*β* production by targeting ubiquitinated inflammasomes for destruction. *Nature Immunology*.

[35] Bandyopadhyay S., Lane T., Venugopal R. (2013). MicroRNA-133a-1 regulates inflammasome activation through uncoupling protein-2. *Biochemical and Biophysical Research Communications*.

[36] Tozsér J., Benko S. (2016). Natural Compounds as Regulators of NLRP3 Inflammasome-Mediated IL-1 *β* Production. *Mediators of Inflammation*.

[37] Han J.-W., Shim D.-W., Shin W.-Y. (2016). Juniperus rigida Sieb. extract inhibits inflammatory responses via attenuation of TRIF-dependent signaling and inflammasome activation. *Journal of Ethnopharmacology*.

[38] Shim D.-W., Han J.-W., Ji Y.-E. (2016). Cichorium intybus Linn. Extract Prevents Type 2 Diabetes Through Inhibition of NLRP3 Inflammasome Activation. *Journal of Medicinal Food*.

[39] Hwang S. J., Kim Y.-W., Park Y., Lee H.-J., Kim K.-W. (2014). Anti-inflammatory effects of chlorogenic acid in lipopolysaccharide- stimulated RAW 264.7 cells. *Inflammation Research*.

[40] Guardia T., Rotelli A. E., Juarez A. O., Pelzer L. E. (2001). Anti-inflammatory properties of plant flavonoids. Effects of rutin, quercetin and hesperidin on adjuvant arthritis in rat. *Farmaco*.

[41] Xia N., Pautz A., Wollscheid U., Reifenberg G., Förstermann U., Li H. (2014). Artichoke, cynarin and cyanidin downregulate the expression of inducible nitric oxide synthase in human coronary smooth muscle cells. *Molecules*.

